# Next-generation sequencing-based detection of germline L1-mediated transductions

**DOI:** 10.1186/s12864-016-2670-x

**Published:** 2016-05-10

**Authors:** Jelena Tica, Eunjung Lee, Andreas Untergasser, Sascha Meiers, David A. Garfield, Omer Gokcumen, Eileen E.M. Furlong, Peter J. Park, Adrian M. Stütz, Jan O. Korbel

**Affiliations:** European Molecular Biology Laboratory, Genome Biology Unit, 69117 Heidelberg, Germany; Department of Biomedical Informatics, Harvard Medical School, Boston, MA 02115 USA; Division of Genetics, Brigham and Women’s Hospital, Boston, MA 02115 USA; Center of Molecular Biology (ZMBH), Heidelberg University, Im Neuenheimer Feld 282, Heidelberg, 69120 Germany; Department of Biological Sciences, State University of New York at Buffalo, Buffalo, NY 14260-1300 USA; European Molecular Biology Laboratory-European Bioinformatics Institute, Wellcome Trust Genome Campus, Cambridge, CB10 1SD UK

**Keywords:** Retrotransposon, L1, Transductions, NGS, Bioinformatics, Genome, Genetics, Primates, Single-molecule sequencing

## Abstract

**Background:**

While active LINE-1 (L1) elements possess the ability to mobilize flanking sequences to different genomic loci through a process termed transduction influencing genomic content and structure, an approach for detecting polymorphic germline non-reference transductions in massively-parallel sequencing data has been lacking.

**Results:**

Here we present the computational approach TIGER (Transduction Inference in GERmline genomes), enabling the discovery of non-reference L1-mediated transductions by combining L1 discovery with detection of unique insertion sequences and detailed characterization of insertion sites. We employed TIGER to characterize polymorphic transductions in fifteen genomes from non-human primate species (chimpanzee, orangutan and rhesus macaque), as well as in a human genome. We achieved high accuracy as confirmed by PCR and two single molecule DNA sequencing techniques, and uncovered differences in relative rates of transduction between primate species.

**Conclusions:**

By enabling detection of polymorphic transductions, TIGER makes this form of relevant structural variation amenable for population and personal genome analysis.

**Electronic supplementary material:**

The online version of this article (doi:10.1186/s12864-016-2670-x) contains supplementary material, which is available to authorized users.

## Background

The completion of the human and of non-human primate reference genome sequences showed that nearly half of the genome is derived from various transposable sequences [1–4]. Due to their ability to move within the genome active retrotransposons represent an important source of genomic structural polymorphisms [5–7]. Retrotransposition involves RNA intermediates inserting via the target-primed reverse transcription mechanism (TPRT) [8]. The TPRT process produces short (4–25 bp) target site duplications (TSDs) at the flanks of the newly integrated elements [9, 10]. Most mobile element activity in humans results from non-LTR retrotransposons including *Alu*, L1 and SVA [11, 12]. Upon transcription, the RNA polymerase may skip weak transcription termination signals (polyadenylation (polyA) signal, 5′-AATAAA-3′ for L1 and SVA), and hence terminate RNA synthesis at a polyA signal further downstream (3′) [13]. This process can lead to 3′ transductions at L1 and SVA elements, causing mobilization of downstream flanking sequence together with the mobile element [14–17]. In addition, short sequence transductions (<50 bp) can occur when the RNA cleavage-prior-to-polyadenylation occurs abnormally and slightly downstream than usual, capturing a small piece of the genomic location after the short poly-A track of the source L1 element [18].

Retrotransposition can contribute to diseases [12, 19] and evolution [12, 20, 21] and previous studies identified differences in the spectrum of mobile element classes among distinct primate species [4, 21, 22]. Transductions play an important role in this process, e.g. through mobilization of genomic functional elements including exons or by resulting in gene disruption events [12, 13, 17, 19, 23–26]. Previous studies focusing on *reference* transductions (i.e. elements present in the reference genome) have reported that transductions are relatively abundant, with estimates that around 10 % of L1 and SVA insertions detectable in the human reference assembly exhibit 3′ transduction events [15–17, 23, 27]. Only few recent studies, however, have investigated *non-reference* transductions and consequently there is little knowledge on transduction-mediated sequences polymorphic in the population. Kidd and co-authors, prior to the widespread application of next generation sequencing (NGS), identified several polymorphic L1-transductions through a fosmid library-based Sanger sequencing approach in nine HapMap samples [28] and MacFarlane and co-authors developed the experimental TS-ATLAS method that uses L1 3′ transductions as sequence tags to identify active L1 lineages in a genome-wide context [29]. Furthermore, more recently, Tubio and colleagues reported an abundance of somatic L1 transduction events in cancer genomes sequenced with short DNA reads [30], Paterson and colleagues identified 3′ transduced sequences in oesophageal adenocarcinomas [31] and two studies recently reported somatic L1 insertions with 5′ and 3′ transductions in human neurons [32, 33] – which highlights that somatic transductions can occur outside of cancer and may be relevant for a broader range of diseases. Detecting variants in somatic genomes, however, is conceptually different from germline polymorphism inference, and polymorphic transduction events arising in germline genomes have – to the best of our knowledge – not systematically been studied by NGS thus far.

Here we describe a computational approach suitable for the discovery of non-reference polymorphic (or monomorphic) mobile element transduction events – termed TIGER for Transduction Inference in Germline genomes – based on Illumina NGS data. We applied TIGER to the detection of L1 mediated 3′-transductions, the most abundant class of mobile element transductions [15, 16], in five chimpanzee, five orangutan and five macaque [21] samples sequenced to a mean coverage of ~20x as well as to the well-characterized human NA12878 lymphoblastoid cell line [34]. Furthermore, we performed extensive experimental validation and event characterization by PCR and state of the art single-molecule long DNA read sequencing technologies. Our analyses demonstrate differences in the rate of transduction across primate species, and highlight species-specific mobile element subfamilies involved in L1 transduction. TIGER, made available open source (http://www.korbel.embl.de/software), makes a relevant class of structural variation amendable for personal genome analysis.

## Methods

### Whole-genome sequencing data

Using TIGER we analyzed previously published chimpanzee, orangutan and macaque whole-genome sequencing (WGS) data [21] from five individuals per species, sequenced between 14.4-28.8x, as well as the human NA12878 sample down-sampled to ~20x (two technical replicates) [34]. Details on read mapping and filtering are in the Supplementary Methods (Additional file 1).

### TIGER specifications

TIGER uses a combination of (1) non-reference L1 insertions – in this study discovered by a modified version of TEA [35], including lower-confidence L1 elements inferred by TEA, to allow for increased sensitivity (see Additional file 1: Supplementary Methods for details) [21], (2) translocation (TL) calls identified using the DELLY [36] translocation detector module as well as (3) single-anchored (SA) reads obtained directly from BAM (Binary Alignment/Map) files. SA and TL reads are found as discordantly mapped read pairs, either having one read unmapped or placed randomly due to the mapping ambiguity (SA), or both reads in a pair mapped onto two different chromosomes (TL) [37]. Overlap between non-reference L1 insertion and TL reads is used as evidence by TIGER to infer the presence of L1-mediated transductions. The search space of each insertion locus was increased by 500 bp on either side (±500 bp) to define the candidate region. Each discordant (TL or SA) read mapping onto L1-mediated transduction candidate regions was obtained and respective mates realigned onto the corresponding reference genome to identify possible element sources (Additional file 1: Figure S1). This additional realignment step was carried out using BLAT [38] (see Additional file 1: Supplementary Methods for more details). At least 50 bp of each realigned TL or SA mate (roughly 50 % of length of the Illumina reads) was required to ensure robust mapping to the reference genome. Furthermore, realigned mates were processed based on the highest bit-score and the total number of possible matches (TM) to find the best reference match and to differentiate repetitive regions (high TM) from unique regions (low TM), respectively. We required clustering of at least four DNA sequence reads (mean size: 101 bp) on the same source chromosome, which enabled us to construct extended sequence stretches that better reflect the portion of unique DNA sequence transduced, whereas clusters of repetitive reads mapping randomly multiple times in the genome were used to infer L1 presence. In line with the sequencing coverage of our data, as well as our observations from manual inspections and experimental validations, the upper limit of clustered reads at one source locus was calibrated to the value of 30, in order to bypass regions containing solo repetitive mobile elements and regions exhibiting a remarkably high number of supporting reads (indicative for poorly assembled regions or genomic regions bearing unrecognized segmental duplications). To ensure that predicted transduction sequences are unique, the mean of all read-specific TM values per locus was set to be ≤3. Once all aforementioned steps were satisfied, the longest possible stretch of each unique source locus was extended by utilizing reads clustering in an overlapping fashion, and without gaps and reported as the computationally predicted transduced sequence.

**Fig. 1:**
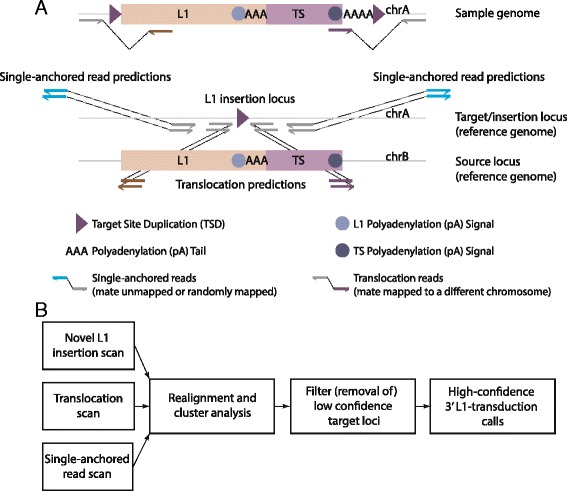
TIGER approach. **a** L1-mediated transduction insertions are typically composed of flanking target site duplications (TSDs, purple triangles), L1 sequence and unique transduction sequence (TS) followed by a non-reference polyA tail. To detect such events in paired-end NGS data, candidate regions are chosen based on an overlap between L1 insertion loci, paired-ends indicative for an insertion of unique sequence copied from a distal locus (as evident from translocation (TL) supporting read pairs), and remapped single-anchored (SA) reads in the reference genome. **b** A combination of reads indicative for L1 insertion as well as unique duplicative sequence insertion and additionally single-anchored reads are used to discover L1-mediated transduction insertions. TL and SA read pairs are realigned to ensure correct placement onto the reference genome. Additional filtering steps are implemented for removal of low-confidence calls

All predicted L1-mediated transduction insertion regions were filtered for overlap with corresponding segmental duplications (using the standardized non-human primate segmental duplication dataset from Gokcumen et al. [21]) as well as the presence of a reference L1 at the insertion to avoid false positives (Fig. 1b). TSDs were extracted directly from the annotation of previously detected L1 elements (identified by TEA [35]), whereas the putative presence of a polyadenylation tail (polyA tail) was evaluated by searching for six consecutive non-reference A′s or T′s (AAAAAA/TTTTTT) in each read.

### L1 subfamily assessment

To assess which subfamily class drives L1 insertions as well as L1-mediated transductions, consensus sequences for all full-length (>6 kb) primate L1s were constructed from multiple reference elements (see Additional file 1: Supplementary Methods for details). To discover L1 subfamilies driving the transduction, longer contig sequences assembled from short reads were realigned to the primate-specific L1 consensus sequences. A best mapping criterion was used to infer the most probable L1 subfamily involved in transduction at each locus.

### PCR and MinION based experimental validations

Experimental validations of L1-mediated transduction predicted loci were performed using PCR coupled with capillary sequencing, as well as by single molecule long DNA read sequencing (see detailed Additional file 1: Supplementary Methods). Human TIGER calls were validated using a PacBio whole genome sequencing dataset of NA12878 [39]. Oxford Nanopore MinION data was generated based on long-range PCR amplicons, using a sequencing platform that we acquired as part of the MinION Early-Access Programme.

## Results

### Computational discovery of L1-mediated transductions through TIGER

TIGER scans genomic regions for the presence of three mobile element transduction-defining signatures: (1) characteristic hallmarks of mobile element insertions (MEIs), including non-reference polyA tails and TSDs, which are retrieved from the Transposable Element Analyzer (TEA) algorithm [35]; (2) aberrant mapping of paired-ends indicative of the adjacent (duplicative) insertion of an additional unique sequence originating from a distal locus (through adaptation of the inter-chromosomal rearrangement discovery module of the DELLY tool [36]); (3) single-anchored paired-end reads (i.e., read-pairs exhibiting an unmapped read or read mapping to repetitive sequences preventing unambiguous read placement) that are reassessed by TIGER to further substantiate insertion signals (see Fig. 1a and Additional file 1: Supplementary Methods for details). The modular nature of TIGER enables it to be applied with any tool for polymorphic mobile element insertion detection. Subsequently, TIGER pursues additional event characterization steps including realignment, read clustering and filtering to identify high confidence transduction events (Fig. 1b and Additional file 1: Supplementary Methods for details).

**Fig. 2:**
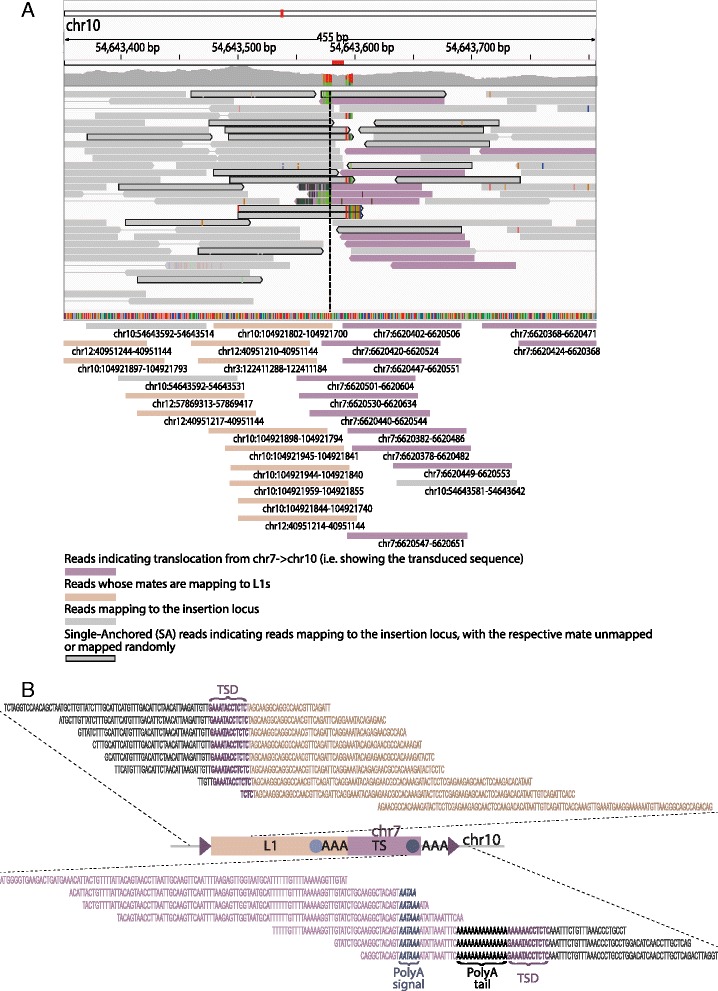
Computational analysis of the chr7:6620368-6620628 insertion into the chr10:54643580-54643593 region in the chimpanzee sample PR01171. **a** Depiction of the chr10:54643580-54643593 region using the Integrative Genomics Viewer (IGV) [57] before read realignment (upper panel). After realignment using BLAT many initially single-anchored reads were placed correctly, facilitating the ascertainment of this L1-mediated transduction clustering to a region on the source chromosome 7 with an average uniqueness of 1 (reads mapping exactly once to the reference genome). **b** A detailed view of L1-mediated 3′ transduction read placements: one read is shown to map to the target locus on chromosome 10 and the other read (mate of the pair) maps either to a non-reference L1 element (displayed on the top panel) or forms a cluster of reads uniquely mapping to the source on chromosome 7 (displayed on the lower panel). Out of 29 reads, 7 were carrying parts of a non-reference polyA tail (only subset of reads shown)

To test TIGER’s utility for detecting polymorphic L1-mediated 3′ transductions, we applied the tool to fifteen recently published genomes from three non-human primate species (five chimpanzees, five orangutans and five rhesus macaques) [21]. An example transduction event detected by TIGER is depicted in Fig. 2. This event involves an inter-chromosomal duplicative insertion of a unique sequence of chimpanzee chromosome 7 (chr7:6620368-6620628) into the respective target region (chr10:54643580-54643593; breakpoint defined by the TSD at the insertion site) mediated by an L1-driven transduction. By realignment onto the reference genome with BLAT [38] we placed previously unmapped (i.e. randomly mapped, or one-end confidently mapped) reads onto the reference genome facilitating characterization of the L1-mediated transduction, as visualized in Fig. 2a. A more detailed view of read placements is provided in Fig. 2b, with one read mapping to the target locus on chromosome 10 and the other read (mate of the pair) either mapping to a non-reference L1 element (displayed on the top panel) or forming a cluster of reads uniquely mapping to the source on chromosome 7 (displayed on the lower panel). Some of these latter read mates contain the non-reference polyA tail and target site duplication (TSD). The additional polyA signal (AATAAA), causing termination of the transduced sequence during the transcription process, is also visible in the data (Fig. 2b). We additionally evaluated the sensitivity of TIGER for predicting 3′ L1-mediated germline transductions in NGS data by performed *in silico* simulations (see Additional file 1: Supplementary Methods for details), estimating a sensitivity of 86 %.

### Experimental verification of transductions by PCR and capillary sequencing

**Fig. 3:**
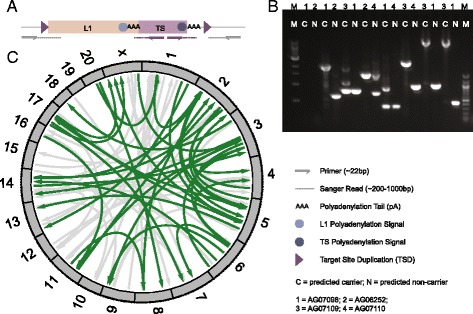
Experimental verification of TIGER-based L1-mediated 3′ transductions by PCR. **a** General primer design: outer (*grey arrows*) primers were placed outside of the event in the target locus to amplify the L1-mediated sequence transduction insertion allele and/or the reference genome allele. On the left side of the locus, the corresponding sequence (*dotted line*) uniquely matches the target site, and subsequently matches to multiple positions in the genome in line with the presence of an L1 element. Further to the right, the sequence will also match uniquely to the target site and end with a polyA stretch not seen in the reference genome. In order to confirm the presence and origin of the transduced sequence (source locus), we employed a 2nd set of primers (*purple arrows*) inside the predicted unique transduction sequence. **b** Example PCRs verifying rhesus macaque L1-mediated sequence transductions, based on outer primers, are shown for inferred carrier (C) and non-carrier (NC) samples. In the presence of an L1-mediated transduction sequence insertion, a larger band than the reference band in NC is seen; heterozygotes show both bands whereas homozygous L1-mediated sequence transduction insertions show only one (i.e. the higher) band. **c** A Circos plot shows the distribution for all inferred rhesus macaque L1-mediated sequence transductions (for orangutan and chimpanzee, see Additional file 1: Figure S6); experimentally validated insertions by PCR and MinION single molecule sequencing are depicted in green. Arrowheads indicate directionality towards the target locus

To verify the accuracy of TIGER, we performed validation experiments on 51 randomly chosen 3′ transduction calls (seven in chimpanzee, 28 in orangutan and 16 in macaque), using PCR followed by capillary sequencing (Table 1 and Fig. 3). We employed a combination of an outer and inner primer pair to specifically amplify the target region, and to overcome the barriers brought about by the two respective polyA tails for pursuing validation by capillary sequencing (Fig. 3a). This validation strategy enabled verification of both the presence of the MEI and of the transduced unique sequence. A representative PCR gel picture for macaque, using outer primers, is shown in Fig. 3b. A Circos plot depicting all predicted transductions in macaque (and in highlighted form with available PCR validation data) is provided in Fig. 3c. In total, we verified 43 out of 51 L1-mediated transduction calls, based on which we estimated a False Discovery Rate (FDR) (see Additional file 1: Supplementary Methods for explanation on FDR calculation) of 15.7 % (with similar FDR estimates across different primate species; Table 1). Investigation of the experimental data on the eight false positive loci revealed that seven lacked MEIs (L1 insertion negative calls) as well as the transduced sequence, whereas the remaining locus presented evidence of an L1 insertion but lacked the inferred transduced sequence (transduction negative call).

**Table 1:** Summary of TIGER results in non-human primate species

Species	Sample	Physical coverage (X)	Non-reference L1 insertions	TIGER transductions	L1 transduction rate (%)*	PCR validated transductions
Macaque	AG06249	26.0	449	29	5.5 ± 1.2**	14/16
	AG06252	29.2	620	28		
	AG07098	21.7	424	26		
	AG07109	23.7	473	28		
	AG07110	18.6	635	28		
Orangutan	AG06105	19.2	663	52	8.8 ± 1.4**	24/28
	AG06209	24.2	803	81		
	GM04272	24.0	649	62		
	PR00054	23.3	775	70		
	PR01110	17.2	633	47		
Chimpanzee	PR00226	32.2	214	4	2.5 ± 1.1**	5/7
	PR00738	32.9	246	7		
	PR00818	28.2	223	4		
	PR01106	19.8	148	3		
	PR01171	18.8	132	5		

For comparison to NA12878 see Additional file 1: Table S5

*Determined based on ratio between TIGER transductions and L1 insertions. 95 % confidence intervals were calculated using one sample *t*-test

**Significantly different based on Wald test of predicted-transduction rates: chimpanzee-macaque: *P* = 0.000073; chimpanzee-orangutan: *P* = 0.000037; macaque-orangutan: *P* = 0.0003

TIGER can be used for estimation of the size of the transduced sequence – however, this capability is strongly dependent on read length and insert-size of the paired-end NGS library, with short NGS reads being only of limited use for detecting the boundaries of the L1 element’s 3′ and the transduced sequence’s 5′ at target loci. In our analyses, sizes of computationally inferred transduction sequence lengths varied between 90–260 bp in chimpanzee, 74–437 bp in orangutan and 64–361 bp in macaque. This suggests that TIGER’s minimal sequence requirement for reliably inferring transductions in Illumina sequencing data is ~50–60 bp.

### Verification and characterization of transductions by single molecule sequencing

**Fig. 4:**
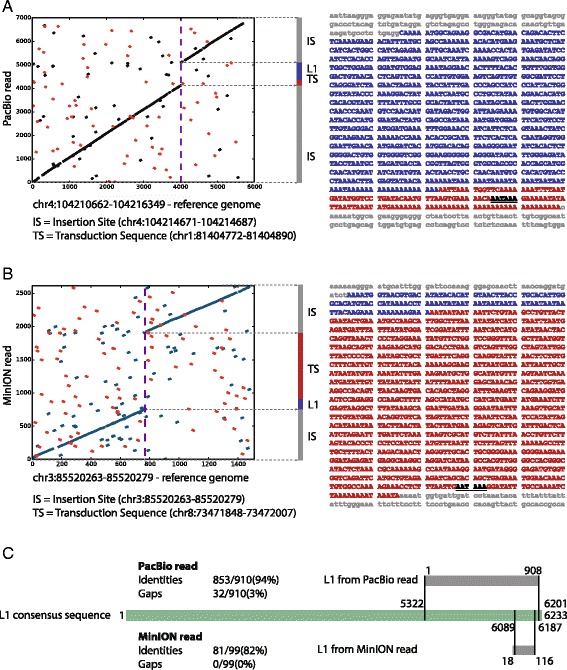
Pacific Biosciences (**a**) and Oxford Nanopore MinION (**b**) long read verification of L1-mediated transduction insertions. **a** *Left panel*: alignment dotplot – surrounding reference genome sequence for the human chr4:104210671-104214687 region shown on the x-axis; PacBio read on the y-axis: ~1000 bp shift shows presence of insertion. Right panel: Inspection of the inserted sequence verified the presence of the L1 element (in *blue*) and the transduced sequence including the new polyA tail (in *red*; based on the consensus sequence created from all PacBio reads); the new polyadenylation signal is underlined. **b** Dotplot – with reference genome sequence on the x-axis and MinION read on the y-axis: ~1200 bp shift shows presence of an insertion. The inserted sequence verified both the presence of an L1 element (in *blue*) and additional transduced sequence including the new polyA tail (in *red*; based on the consensus sequence created from subset of MinION reads). **c** Alignment of the inserted L1 sequence to the ~6 kb long L1 consensus sequence shows that the integrated L1 is 5′-truncated (pairwise-alignment performed with BLAST)

Both short NGS reads and Sanger sequencing reads do not typically fully span the target locus, which complicates the characterization of transduction events. We reasoned that third generation long-read single molecule DNA sequencing technologies may help overcome this challenge, by fully recovering the complete sequence and structure of the combined insertion. Hence, we employed both Pacific Biosciences (PacBio) sequencing [40] (Fig. 4a and c) and Oxford Nanopore MinION sequencing (Fig. 4b and c) to obtain further insights into L1-mediated transductions.

We first employed TIGER to discover transduction events in the human HapMap DNA sample NA12878 (down-sampled to a similar coverage as the primate data [34]), which enabled us to overlay TIGER transduction calls with long DNA sequencing read data (mean = 2425 bp, median = 4891 bp) previously generated by whole genome sequencing (WGS) using PacBio technology [39]. Our analysis of the data showed that long DNA reads are indeed valuable for characterization of L1 transductions. As an example, alignment of a 7 kb long PacBio read from NA12878 to a transduction candidate locus on chromosome 4 (chr4:104210671-104214687) demonstrated a ~1 kb shift in the alignment, further substantiating the presence of the insertion predicted by TIGER (Fig. 4a left panel). Additional inspection of the PacBio sequence allowed us to characterize the structure of the event in more detail (Fig. 4a right panel and 4c). Indeed, analysis of the inserted sequence verified the presence of an L1 3′ transduction, with a 908 bp 5′-truncated L1 element exhibiting a 126 bp long transduced sequence ending with a polyA tail in 3′. Using the previously published PacBio WGS data for NA12878 [39] as well as fosmid sequencing data generated previously for NA12878 [28], we verified four out of six L1-mediated transductions identified in this human sample (two through PacBio reads and two since they were also present in the Kidd et al. dataset; Additional file 1: Table S1). From the remaining two human events, one showed a solo L1 insertion (lacking a transduced sequence) upon further inspection of the PacBio reads, whereas the other locus remained inconclusive, as it lacked coverage of PacBio reads at the genomic region in question, preventing us from verifying the element by computational means.

Second, we obtained similar validation results for all three non-human primate species, through Oxford Nanopore sequencing data which we generated as part of the MinION Early-Access Programme, following long-range PCR amplification of candidate loci (read length min = 155 bp; max = 8848 bp). For example, MinION reads spanning the rhesus macaque L1 transduction candidate locus on chromosome 3 (chr3:85520263-85520279) verified the presence of a ~1.2 kb insertion (Fig. 4b), and further analysis of the inserted sequence demonstrated a 116 bp long 5′-truncated L1 element and transduction of 1043 bp of additional sequence including the new polyA tail in 3′ (Fig. 4b and c). Overall, we validated 38 transductions by single molecule sequencing (36 by MinION and two by PacBio), which combined with the 45 PCR validations (43 validated random calls in addition to two handpicked macaque calls that we did not use for the FDR calculation) resulted in 83 experimentally validated L1 transductions – to our knowledge the largest dataset on validated non-reference germline mobile element transductions reported to date (Additional file 2).

Facilitated by the generated long read sequencing data we examined the length distribution of the inserted L1 elements and of the transduced sequences, observing transduction sequence lengths ranging from 51 to 1570 bp (see Additional file 1: Figure S2). Our validation experiments further verified an abundance of 5′ L1 sequence truncations as previously reported in a similar context [20, 30, 41]. Among 81 experimentally validated transductions, most showed relatively small L1 elements (with only five containing an L1 element >5 kb at the insertion locus).

### Investigation of transduction rates in primate species

We further made use of inferred transduction events to estimate rates of transduction in different non-human primate species, encouraged by earlier studies demonstrating differential activities of solo mobile element insertions across primate species [4, 21, 22]. TIGER altogether detected 274 (266 polymorphic) non-redundant L1-mediated 3′ transductions in the 15 primate genomes, 71 in rhesus macaque, 191 in orangutan and 12 in chimpanzee (Table 1 and Additional file 2). Average numbers of L1-mediated transductions per individual were 27.8 for macaque, 62.4 in orangutan and 4.6 in chimpanzee.

To calculate the rate of transductions per species, we divided the number of high confidence TIGER transduction calls by the total number of non-reference L1 insertions (including solo L1s and transducing L1s) identified using TEA [35] or TIGER (Additional file 1: Figure S3). Our transduction rate estimates were significantly different between species with estimates of 2.5 % ± 1.1 CI (*t*-test, 95 % confidence intervals) for chimpanzee, 8.8 % ± 1.4 for orangutan, and 5.5 % ± 1.2 for macaque, (Wald test of predicted-transduction rates: chimpanzee-orangutan (*P* = 0.000037), chimpanzee-macaque (*P* = 0.000073) and orangutan-macaque (*P* = 0.0003), Table 1).

We further tested whether the observed difference in transduction rates among species could reflect underlying differences in the efficacy of selection among non-human primates with different effective population sizes. We observed no evidence that selective constraints varied substantially among these primate species, and obtained little evidence for an impact of effective population size on the efficacy of selection (see Additional file 1: Supplementary Methods and Additional file 1: Table S2).

The total amount of L1 calls in the human NA12878 down-sampled genome was 79 (after necessary filtering to obtain a high-confidence L1 prediction callset; See Additional file 1: Supplementary Methods for details) – resulting in a transduction rate estimate of ~7.5 % (6/80; of six transductions, five of them are found among 79 L1 calls and one of them is not). Similarly, filtering the Kidd et al. data [28] for calls overlapping with L1 elements in the target regions (events that would not be unambiguously mappable with Illumina reads) and requiring more than 50 bp of uniquely mappable transduced sequence, resulted in an adjusted transduction rate estimate of 10.8 % (see Additional file 1: Supplementary Methods for details as well as Additional file 1: Table S3).

Lastly, we examined whether TIGER detected 5′ transductions in our primate dataset. Notably, we did not observe evidence for a single L1 5′ transduction event driven by a new upstream promoter, which is consistent with earlier reports based on reference transduction analysis suggesting a very low rate of such events [42, 43].

### Characterization and L1 subfamily analysis of transduction events

To investigate potential functional consequences of transductions we analysed the overlap of transduced sequences (based on their source and target coordinates) with annotated functional regions of the genome, considering all events identified in this study. The majority of transduction sequences were derived from intergenic regions (205/280) and a similar fraction also inserted into intergenic regions. Approximately a third (90/280) inserted into intronic regions, where some may have an effect on gene regulation [19, 35, 44] (Additional file 3). Intersection of source coordinates of inferred transductions with exonic annotations, furthermore, identified two candidate events – one in orangutan and one in macaque. In both cases, while there was strong evidence for the insertion of unique genomic sequence, evidence for L1 associated sequence signatures was minimal. Notably, following further manual inspection and PCR validation, both insertions turned out to represent gene retrocopy insertion polymorphisms (GRIPs) [45, 46] rather than transduction events. GRIPs share many diagnostic features with transductions, such as a TSD and the insertion of unique sequences, including the presence of a polyA tract, as they are mobilized by the L1 machinery *in trans* [45], which may explain why TIGER was able to identify these events in this context (Additional file 1: Table S4).

We further investigated the source-donor L1 relationship with a focus on transduced sequences. Among three out of the six transduction calls observed in humans, we obtained evidence for a full-length reference L1 (specifically human-specific L1HS) element immediately upstream of the predicted transduction source locus (Additional file 1: Figure S4). Contrary to the human genome, we saw no clear indication confirming the presence of full length donor L1 elements at source loci within non-human primates, apart from one source region in rhesus macaque exhibiting a >5 kb long L1PA7 element within a 10 kb region surrounding the transduced source sequence. We therefore classified the source loci of all validated transductions into two classes: (1) no donor L1 fragments annotated within 5 kb to either side of the transduced sequence (L1 elements segregated differently from the target site in the population) and (2) presence of small, truncated L1 elements surrounding the transduced sequence, either unrelated to the transduction or severely truncated following the formation of the transduction event (Additional file 1: Figure S4 and Additional file 4). Our analysis suggests that the majority of calls belong to class (2), i.e. 22 in rhesus macaque, 30 in orangutan and five in chimpanzee, whereas the remainder fall into class (1) (13 in rhesus macaque, nine in orangutan and one in chimpanzee). In addition, our analysis did not reveal any hotspot donor L1s generating multiple transductions, as recently described in an in-depth analysis of somatic transduction events in cancer genomes [30].

**Fig. 5:**
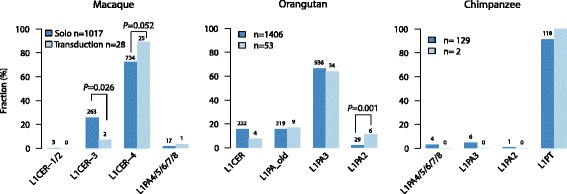
L1 subfamilies associated with L1-mediated transductions: *P* values are based on Fisher’s exact test per subfamily using 2 × 2 contingency tables

Finally, we investigated L1 subfamilies responsible for transductions to evaluate whether L1 subfamily specificity may explain the differential rate at which L1 elements are accompanied by transduced sequences in different species (see Table 1 and Additional file 1: Table S5). To this end we remapped contigs assembled from short reads aligning to the inserted L1 sequence to the consensus L1 subfamilies and enumerated best alignment hits (i.e., reads showing fewest mismatches; see Fig. 5, Additional file 1: Figure S5 and Additional file 1: Supplementary Methods). Notably, 96 % of all annotated L1-mediated transduction insertions in rhesus macaque belonged to the L1CER family (notably L1CER4), which has evolved from macaque-specific L1PA6 [47]. In orangutan, by comparison, 63 % of the annotated L1-mediated sequence transductions were associated with the L1PA3 subfamily and 92 % with any L1PA family member. Furthermore, in chimpanzee all transductions were found in association with L1Pt members. Interestingly, we observed examples where L1s accompanied by transductions showed a different subfamily distribution than solo L1s in the respective species (Fig. 5 and Additional file 1: Figure S5). In macaque, for example, L1CER-4 showed a slight enrichment for transductions (*P* = 0.052), albeit not nominally significant, whereby L1CER-3 associated transductions showed depletion relative to solo L1s (*P* = 0.026). By comparison, in orangutan L1PA2 elements exhibited an increased rate of transductions relative to solo L1s (*P* = 0.001), whereby in chimpanzee too few L1 transductions with reliable subfamily annotation were identified to allow for robust statistics.

## Discussion

Retrotransposon mediated transductions are an important class of polymorphic structural variation in the germline, which so far has been largely unexplored, and TIGER renders this class of genetic variation amenable to NGS-based analyses. While the detection of transductions presents technical challenges in short read DNA sequencing data, owing to the repetitive nature of mobile elements [48] and the fact that Illumina sequencing reads are short when compared with L1 sequences, we have demonstrated TIGER’s ability to robustly identify L1-transduced sequence elements. Our data indicate variability in transduction rates between species, with rhesus macaque and chimpanzee exhibiting significantly reduced transduction rates (5.5 % and 2.5 % of all ascertained L1 events, respectively) compared to orangutan (8.8 %), which adds to previous findings of differential activities of mobile elements among primate species [4, 21, 22]. We note that a number of L1 insertions associated with transductions in the samples covered by our study were previously overlooked in a scan for solo L1 insertions [21], presumably since in the presence of a transduction these events lacked sufficient evidence from both 5′ and 3′ flanks for solo L1 inference – which indicates that application of TIGER can increase sensitivity for L1 detection. Differences in transduction rate may have evolutionary consequences, given that transductions can mobilize functional genomic DNA sequences [17]. These differences may at least in part be mediated by L1 subfamily usage.

Our transduction rate estimate for humans (7.5 %) is slightly lower when compared to previous studies reporting ~10 % [15, 17, 49], a difference that may be attributed to the stringent filtering we performed. Kidd et al. [28] identified transductions accompanying 20 % of all non-reference L1s, resulting in a higher transduction rate – but when we employed equivalent filters used in conjunction with TIGER on the Kidd et al. data, we obtained a comparable transduction rate estimate of 10.8 %. It should be stressed that we designed TIGER for the analysis of short (Illumina) NGS reads, which are known to map ambiguously in the context of repetitive genomic sequence when compared to PacBio or capillary sequencing reads – and remaining limitations related to the use of Illumina data exist across methods utilizing short reads. Thus TIGER may be insensitive to elements that insert into or derive from regions of low mappability [7, 50], as well as to transductions <50 bp in length. Another limitation of our study is that we did not specifically investigate orphan transductions arising from the same process [30].

It is possible that an improved FDR of TIGER may be achieved in conjunction with higher confidence MEI calls. In this regard, our FDR estimate for TIGER (15.7 %) is well in line with a recent FDR estimate (16–24 %) for the TEA mobile element discover algorithm [35] used for inferring MEI signals in our study [51]. Furthermore, our investigation of PacBio and MinION single molecule DNA long read sequencing data demonstrates the potential of third generation sequencing for uncovering such events, with the promise to facilitate identification of these also in more repetitive regions of the genome as already suggested in a study by Chaisson et al. [52], once such technologies are more widely applied at a genome-wide scale.

Although TIGER should in principle be capable of identifying 3′ and 5′ transductions accompanying L1 insertions, we have not observed a single 5′ transduction event driven by an alternative upstream promoter in our data. 5′ transduction events were shown to be extremely rare in human genomes with only few cases reported [32, 42, 43]. Scarcity of 5′ L1 transductions may also relate to the common truncation of the 5′ part of the transcript (normally the L1 element) observed during the TPRT based integration mechanism, which would be expected to particularly affect 5′ transduction sequences upstream of the L1 element. A recent study of somatic L1 transduction events in cancer genomes is consistent with a scarcity of 5′ L1 transductions [30]. Cancer-associated transductions, furthermore, have been reported to be typically highly clustered, whereby a single source L1-master element tends to cause several transduction events in unrelated samples [30]. Interestingly, we did not observe such clustering in the samples studied here, perhaps due to relaxed suppression of active L1 elements in the germline, which may reduce event clustering. In somatic tissues most of active L1 sources are suppressed, and clustering may occur when one or few escape from the suppression, mediated, for example, by local alterations in chromatin structure and DNA methylation in cancer cells.

Our study focused on the inference of polymorphic L1 transductions, with L1 elements belonging to the autonomous retrotransposition-competent mobile element class commonly mobilizing non-repetitive sequences. While SVA elements are also capable of transducing unique DNA sequences [17] and therefore could be a future scope of further TIGER development, they are still absent from the macaque genome [53], and previously observed novel non-reference SVA elements in other species were relatively small in number [21]. *Alu*-mediated transductions have so far not been reported in the literature and thus were not a target of our study. In principle, while we chose to work with the TEA algorithm to identify L1 input signals, TIGER can be used in conjunction with other MEI discovery algorithms, including Tangram [54], Mobster [55], TE-Tracker [56] and RetroSeq [51], augmenting the functionality of these existing tools. By mobilizing unique sequences, L1 elements can shuffle and duplicate potentially functional genomic segments adding to genomic diversity. TIGER enables investigation of this relevant layer of genetic diversity in personal genomes, with potential future applications to disease and evolutionary studies.

## Conclusions

We developed TIGER for identifying non-reference retrotransposon-mediated transductions in the germline using NGS data. TIGER, which can be used in conjunction with a number of translocation and non-reference retrotransposon discovery tools, will enhance variant analysis pipelines, and offers access to an as yet under-explored type of germline genetic variation.

### Ethics and consent to participate

Not applicable.

### Consent to publish

Not applicable.

### Availability of data and materials

All data presented in this study is either a part of the manuscript or supplementary files listed below.

## Additional files

Additional file 1: Contains all supplemental material and methods and supplemental figures and tables. (PDF 1089 kb) Additional file 2: Is a list of all L1-transductions discovered in this study and their validation status in macaque, orangutan, chimpanzee and human. (XLSX 109 kb) Additional file 3: Contains a list of all L1-transductions discovered in this study near genes that either originate from an intronic sequence or insert into an intronic sequence in macaque, orangutan, chimpanzee and human. (XLSX 63 kb) Additional file 4: Contains IGV representations from each experimentally validated source locus (two representations: 5 and 10 kb surrounding each source locus). (ZIP 46533 kb)

## Abbreviations

FDR: false discovery rate
GRIP: gene retrocopy insertion polymorphism
ME: mobile element
MEI: mobile element insertion
NGS: next generation sequencing
pA/polyA: polyadenylation
SA: single-anchored
SV: structural variant
TL: translocation
TS: transduced sequence
TSD: target-site duplication
