# Supplementary material for: Next-generation sequencing-based detection of germline L1-mediated transductions
Source: BMC Genomics. 2016 May 10;17:342. doi: 10.1186/s12864-016-2670-x (PMC4862182; doi:10.1186/s12864-016-2670-x)
Supplement: Additional file 1: — Contains all supplemental material and methods and supplemental figures and tables. (PDF 1089 kb) [file 12864_2016_2670_MOESM1_ESM.pdf]

SUPPLEMENTAL MATERIAL AND METHODS

Data used in this study

The TIGER tool was applied to 25x WGS data from three different non-human primate species – chimpanzee, orangutan and macaque (five individuals per species, sequenced between 14.4-28.8x, [1] and a human sample NA12878 (HapMap/1000GP CEU daughter from ftp://ftp.1000genomes.ebi.ac.uk/vol1/ftp/ [2]), downsampled to ~21x using two independent technical replicates (by Downsample.jar from PicardTools version 1.52 (http://picard.sourceforge.net.) using either a predefined random-seed value or a default random seed value to create two technical replicates). Similar numbers of transductions and MEIs were identified upon analysis of each replicate. All DNA reads were sequenced using the Illumina HiSeq platform, using 101 bp paired-end reads.

The WGS data was aligned onto the corresponding reference genome builds: human hg19, chimpanzee panTro3, orangutan ponAbe2 and rhesus macaque rheMac2 using the Eland v2 software from Illumina for the non-human primate species data and BWA [3] for the human data. Non-human primate ME calls were determined using an extended version of TEA (see 'Non-reference L1 insertion discovery', [4]). Due to the TEA tool requirements, non-human primate data was remapped with BWA in order to detect non-reference L1 calls. All L1 calls (including low-confidence L1 calls that may lack support from either the 5' or 3' end of the L1 due to the presence of a transduced sequence) were considered as a seed for possible transduction events. For each species, translocation calls were inferred using Delly v0.0.11 (jumpy_v0.0.11) [5].

Non-reference L1 insertion discovery

The TEA pipeline [4] was used to perform non-reference MEI discovery. TEA detects an MEI by identifying 1) clusters of 'repeat-anchored mate' (RAM) reads, which are uniquely mapped to the reference genome and have paired mates that map to a ME sequence library, and 2) partially-aligned reads spanning the insertion breakpoints ('clipped reads'), whose unaligned tail sequences match the inserted ME. The ME sequence library was built by concatenating multiple consensus sequences of ME subfamilies separated by 200 'N' nucleotide spacers. For L1, consensus sequences for L1HS, L1PA3, L1PA5, L1Pt were used. To facilitate detection of L1 events exhibiting transductions, we also included RAM clusters having RAMs appear only on one side of the insertion candidate (low confidence TEA calls). At least three RAMs and at least one clipped read on either or both sides of an insertion was required in order to detect L1 events with transductions. Whenever needed for comparative purposes, we made use of the high-confidence dataset of solo-L1 events released by Gokcumen *et al.* [1].

In the human genome (NA12878) analyzed, L1 element filtering we applied was comparable to how we filtered the primate data [1]: target loci overlapping with any L1 elements, including evolutionary older L1 subfamilies were filtered out. In contrast to recent somatic retrotransposon studies by Tubio *et al.* [5] and Lee *et al.* [4], which filtered target regions annotated with evolutionary young L1 elements only, our survey critically requires more stringent filtering as we are assessing fairly old L1 elements, such as L1PAs in primate genomes, too.

Computational prediction of L1 transduction candidates by TIGER

To identify candidate L1-transduction insertion events, intersectBed from BEDTools [6] was used to obtain intersections between non-reference L1 insertion coordinates and intervals inferred to be supporting a translocation (TL) using the DELLY algorithm's translocation detection module. We increased the search space by 500 bp on either side of the L1 insertion (±500 bp) to avoid undercalling intersections, with L1 insertion being typically demarcated by coordinates of short TSD sequences. In addition, this maximizes the usage of paired-end reads from NGS libraries which have a typical insert size of 300-500bp. To further increase sensitivity, TIGER additionally looks for TL and single-anchored (SA) paired-end reads for which one read (end) maps to candidate regions (±500 bp) directly from the BAM file [7]. We performed realignment of each mate paired with individual read to the corresponding reference genome using the UCSC standalone BLAT software (version 34) [8] to either confirm (with TL reads) or help infer (with SA reads) the source chromosome. While in the case of TL reads both ends are (by definition) already mapped in the BAM file, mapping of such reads sometimes creates artifacts, especially if the read sequence aligns to repetitive regions of the genome including L1 elements – hence usage of the BLAT-based realignment step to confirm read placement.

BLAT [8] hits were further subjected to further filtering based on the highest bit-score to find the highest confidence reference match of all possible matches. Additionally, the total number of possible matches (here abbreviated as TM) was recorded, allowing to distinguish repetitive from unique regions in the genome (i.e. repetitive regions will have relatively high TM due to their mapping to multiple locations in the genome, when compared with unique regions). In addition, only reads mapping with at least 50 bp to the reference genome were taken into consideration (alignment length from BLAT output [8], AL=50 bp, that is a minimum of ~50% of length of the Illumina reads).

For each valid candidate insertion locus, we required at least 4 paired-ends supporting the presence of a transduced sequence, whereas the upper limit of clustered reads at one source locus was calibrated to the value of 30.

Importantly, in order to increase the confidence in the transduction support, the longest stretch of the source loci possible was assembled by using only reads that clustered in an overlapping fashion (i.e. gaps between reads were not allowed). Finally, we removed events overlapping with a segmental duplication (using the combined segmental duplication dataset used in Gokcumen *et al.* [1]) or a reference L1 element at the candidate locus (a commonly used filter in non-reference mobile element detection [4, 9]), to remove possible artifacts (Supplementary Figure 1).

To annotate and further characterize candidate transduction events, TSD values were directly extracted from the TEA output [4] whereas the presence of a putative polyadenylation tail (polyA tail) was evaluated by searching for six consecutive non-reference A's or T's (AAAAAA/TTTTTT).

During earlier stages of the project we also attempted to identify evidence for *Alu* and SVA based transductions but failed to identify high-confidence events, suggesting that such classes of transductions are either exceedingly rare or absent.

In silico TIGER simulations

In order to measure the sensitivity of TIGER, we simulated transductions based on previously identified transduction source elements into the orangutan reference genome (ponAbe2). To achieve a wide representation of transduced sequences we used the set of 198 transductions, including 39 validates loci previously inferred in orangutan by TIGER, ranging from 74bp to 437bp in size. For each locus we randomly and uniformly picked a position in the reference genome (ponAbe2) allowing only chromosome 1-22 and X and excluding assembly gaps (stretches of N) and regions 1kb upstream and downstream of these gaps. Transduction events that we randomly inserted consisted of a short sequence from the target site (target site duplication, TSD), an L1 sequence, the transduced sequence, as well as a poly-A tail followed by the TSD. Since for many of the reported transductions we did not observe an L1 element in the reference genome flanking the source site, we employed the full length L1 consensus sequence for generating these random insertions. The length of the TSD was randomly sampled from the distribution described in Helman *et al.* [10], but we excluded negative values, zeros and outliers >50bp (median: 15bp). The poly-A lengths were sampled from Solyom *et al.* [11] and are in the range of 11-104bp with a median of 32bp.

We created a modified version of the reference genome carrying 198 simulated transductions, and subsequently *in-silico*-sequenced this genome using WGSIM (https://github.com/lh3/wgsim) with 2x100bp reads, an insert size of 300bp (standard deviation 60) and a sequencing coverage of 17x, matching the average coverage used in the primate samples. Reads were mapped back to the original reference using bwa mem [3]. We then executed DELLY [12] (version 0.0.11, same as for the original calls) to call translocations. To evaluate the sensitivity of TIGER we assumed optimal/perfect sensitivity of TEA, creating a TEA output file with mobile element calls mapping to the simulated coordinates (which allowed us to evaluate TIGER irrespective of the sensitivity of the MEI calling step). The whole procedure was repeated in total 5 times, leading to a total of 990 simulated transductions. We then executed the TIGER accessory module, estimating a sensitivity of 86% (853/990). Analysis of the individual runs showed that only three source elements could not be predicted by TIGER consistently across all five runs (98.5% of all tested transduction events were successfully recovered in at least one simulation round), indicating that it is mostly the character of the target region driving sensitivity instead of any of the simulated properties of the transduction or the parameters used by TIGER.

Estimating the subfamily of L1 insertions

To assess which subfamily drives L1 insertions as well as L1-mediated transductions, we assembled sequences of clipped reads and RAM mates into longer contig sequences using CAP3 [13]. L1 subfamily information was annotated based on the sequence similarity of contigs assembled from L1 mapping reads to consensus sequences of different L1 subfamilies, using blastn. Consensus sequences of 42 L1 subfamilies (T1-1D, L1PA1-L1PA8 from RepeatMasker, chimpanzee-specific L1Pt, 32 rhesus macaque-specific L1CER elements [14]) were compiled. After multiple sequence alignment using ClustalW2 (http://www.ebi.ac.uk/Tools/msa/clustalw2/), 922 bp from the 3' end of each subfamily were used for subfamily evaluation. This enabled us to readily compare contig alignments across subfamilies. Since this annotation relies on the information of contigs coming from the ends of the inserted L1, we found that L1s were often assigned to more than one subfamily. Thus, to better utilize the annotation for understanding subfamily distributions of solo and

transduction L1s, subfamilies were collapsed to the major subgroups– for example, we merged 6 L1CER-1 subfamilies (L1CER-1A ~ L1CER-1F) into 'L1CER-1'. The overall subfamily distribution was examined using solo L1s with unique subfamily annotation. Close subfamilies were joined, if one subfamily had too few L1s assigned to it (*i.e.*, when the number of L1s observed was less than 3). For example, L1CER-1 and L1CER-2 in Macaque were merged into one group based on this criterion. Using the revised subfamily groups, we analyzed the subfamily breakdown of both solo and transduction L1s with unique subfamily annotation (Figure 5) and with multi-subfamily annotation (Supplementary Figure 5). When using multi-subfamily annotation, an L1 was assigned to multiple-subfamilies (L1s with < 5 subfamilies were considered) to each subfamily with an equal weight. For example, if an orangutan L1 was annotated with L1PA2 and L1PA3, 0.5 was added to both L1PA2 and L1PA3 for the L1 in the subfamily breakdown.

Design of experimental validations

We employed PCR with several primer combinations as well as capillary sequencing to validate transduction candidates and assess the False Discovery Rate (FDR) of the TIGER tool. For this, we decided to test ~20% of the predicted transduction loci (which were randomly selected with the 'shuf' Unix command). Outer primers were designed to bind to unique regions at least 100bp away of the target integration site, using an in house primer design tool. Furthermore, an inner set of primers (inside transduction sequence) was designed using Primer3Plus [15]. PCR amplicons were amplified with the outer primer set, whereas capillary sequencing was performed with both the outer and inner primers, for each locus.

PCRs were preformed using 10ng of genomic DNA [1] in 25 µl volumes using the Sequalprep Long PCR reagents (Life technologies) in a 96 well plate. PCR conditions were: 94°C for 3min, followed by 10 cycles of 94°C for 10 s, 62°C for 30s and 68°C for 6 min and 25 cycles of 94°C for 10s, 60°C for 30s and 68°C for 8min, followed by a final cycle of 72°C for 10min. PCR products were analyzed on a 0.8% agarose gel stained with Sybr Safe Dye (Life Technologies) and a 100 bp ladder and 1 kb ladder (NEB). If necessary, gel bands were cut with a scalpel, gel extracted and send for capillary sequencing. Sequence chromatograms were manually inspected and sequences were analyzed using BLAT. When aligning sequences generated using outer primers to the reference genome these typically split into a sequence with multiple genome matches (corresponding to the non-reference L1 element), as well as a sequence stretch containing a non-reference polyA/T stretch demarcating the end of either the L1 element or the transduced sequence. To distinguish both possibilities, analysis of the sequences from the inside primer pair was pursued, which should correspond to the transduced sequence and should match uniquely to the source chromosome, on one side flanked by a non-reference polyA/T stretch indicating the end of the transduced sequence and a polyA/T stretch on the other side, indicating the end of the L1 element involved in the transduction event. In a few cases, we obtained unclear results. These were either due to PCR failure (no product despite usage of 2 independent outer primer pairs) or sequencing failure (observation of a band larger than the expected reference sequence, in support for the presence of an insertion, but missing sequence information). For loci were we only detected a reference PCR band (no mobile element related insertion), despite using a 2nd independent primer pair, we concluded that the validation was negative, and used this to calculate the FDR (number of negatives/number of positives+negatives). This FDR estimate we report is likely conservative, as computational inspection of false positive loci in the IGV browser showed credible signs of retrotransposition insertion and transduction in several cases. During the

PCR, the significantly higher band (up to 6 kb+transduction size) corresponding to the allele carrying the insertion is generally disadvantaged. Furthermore, if the DNA quality is not optimal, such large bands might not be efficiently amplified anymore. Nevertheless, we decided to report the conservative FDR based on the experimental findings, as an independent validation strategy for the computational prediction, but mentioning the limitations of the PCR.

Oxford Nanopore MinION library preparation and sequencing

The purified amplicon PCR DNA pool was used with the Genomic DNA Sequencing kit (version SQK-MAP005) for MinION library prep as part of the MinION Early Access Programme (Oxford Nanopore Technologies). Briefly, 1µg of amplicon pool DNA and 5ul of control DNA-CS were end repaired using the End repair module reagents (NEB) for 20min at 20degree, purified with 100µl of AMPure XP beads and eluted in 25µl nuclease free water. A-tailing (NEB) was performed in 30µl for 10min at 37 degree and afterwards cleaned up with 30µl AMPure XP beads and elution into Protein LoBind tubes (Eppendorf). Next, adapter ligation was performed by adding 10ul AMX, 10µl of HPA and 50µl of Blunt T/A ligase mix (NEB) and incubation for 10min at 20 degree. A special cleanup step with 100µl washed His-Tag beads in Protein LoBind tubes was performed, using 2x200µl of BBB wash buffer and elution into 25ul provided elution buffer ELB without a drying step. Briefly before the MinION sequencing run, 6ul of this pre-sequencing mix was mixed with 75µl RNB buffer, 66µl NFW buffer and 3µl of Fuel mix, gently mixed to produce the final library and loaded on a primed MinION flowcell (version FLO-MAP003). MinION flowcells were used with the software client Metrichor Agent v 2.31.1, the sequencing software MinKNOW v 0.50.1.15 and the 2D Basecalling version v1.6. Fastq reads were extracted using the h5py python package and converted to fasta files employing the fastqToFa function of the kent package (http://hgdownload.soe.ucsc.edu/admin/exe/linux.x86_64/FOOTER). The reads were mapped to the putative PCR fragments using lastal version 473 with the parameters -r4 -q4 -a10 -b3 -e400 -m50 -Q0 (LAST, http://last.cbrc.jp). Based on the alignment dotplots were calculated. Mapped reads were analyzed by generating dotplots (pairwise alignment against the PCR amplicon sequence; see also Figure 4), and the sequence of reads indicating an insertion were extracted and further analyzed by BLAT to identify the presence and length of the L1 element and of the transduced sequence. To maximize coverage of informative reads, only PCR bands which were different in size than the reference band were extracted and sequenced with the MinION. Therefore, these positive loci are not included in the FDR calculation of the PCR, but are counted for the total number of validated loci, annotated in the Extended dataset, and the resolved L1 and transduced sequence sizes were used to calculate their distribution.

Wald test of predicted-transduction rates

To test whether the differences between transduction rates were significant, the L1-transduction dataset was fitted to a Poisson linear model with a dispersion parameter (a particular case of a Tweedie model). A log-link was used and the dispersion parameter was estimated using the R functions glm [16] and the implementation of the Tweedie distribution in the package statmod [17].

The coefficients taken into account to fit predicted transduction numbers were: species, number of all high-confidence solo-L1 insertions and physical coverage, to ensure none of the

mentioned coefficients would create a confounding bias. Contrasts between coefficients representing pairwise differences between the species were computed using the glht function from the multtest [18]. Subsequently, Wald tests of the contrasts using a t-distribution with the residual degrees of freedom of the regression model were performed to obtain the *P*-values for every pairwise comparison: chimpanzee-orangutan (*P*=0.000037), chimpanzee-macaque (*P*=0.000073) and orangutan-macaque (*P*=0.0003).

To use the Kidd *et al.*[19] dataset for comparative purposes, we lifted coordinates from the hg18 to hg19 reference build using the liftOver tool from the UCSC Genome Browser (http://genome.ucsc.edu/ [20]). In order for this dataset to be comparable to transduction calls generated by TIGER in our dataset, we required the absence of additional L1 elements and segmental duplications immediately surrounding the target locus. In addition, we excluded transductions where the source was not determined and where the listed transduction sequence was not unique (see Supplementary Table 3 for a list of criteria employed). We similarly filtered the Tubio *et al.* [5] dataset, excluding candidate insertions into reference L1 elements and/or segmental duplications.

Effective population size effect on L1-transduction rate

To assess the impact of effective population size (and thus the efficacy of selection) on L1-transduction rate, we first estimated the genotypes (comprising reference or alternate alleles) at each site in each species. When possible (rhesus macaque and orangutan), genotypes were scored for autosomes and chromosome X separately (all scored L1 transduction events in chimpanzee were autosomal). In our scoring system, non-reference calls are dominant such that individuals with alternate genotypes could be heterozygous or homozygous. As a result, we could not calculate population genetic summary statistics directly from the data. To circumvent this issue, we treated each locus as being in Hardy-Weinberg equilibrium (following Hazzouri *et al.* [21]), and used this assumption to obtain estimates of the frequency of the reference and alternative alleles in the population. We then generated 100 sets of genotypes in which the exact constitution of the ‘alternate’ individuals (heterozygous or homozygous) was determined by sampling from these frequency estimates. For each of the 100 sets, we calculated Tajima’s D, Watterson’s Theta, and Pi using custom python scripts [22]. The mean of these samples as well as the 95% quantiles are recorded in the Supplemental Table 2. It is important to note that in many cases there are fewer than 100 possible configurations for the alternative individual genotypes. Thus, these 95% quantiles cannot be treated as proper distributions of the data.

The observed difference in transduction rates among species may reflect underlying differences in the biology of each species or, more likely, differences in the specific L1 families segregating in the three species. It may also, however, reflect different selective pressures against L1 transduction events. Research in multiple systems suggests that transposable element (TE) insertions are often deleterious [23, 24], and that the distribution of TEs in natural populations can be best explained by a model that treats selection on TEs as a steeper-than-linear function of TE copy number [25]. In the context of these models, differences in the distribution of TEs among species can be explained by differences in transposition rates or by differences in effective population size (N_e), which influences the relative strengths of selection and drift [26].We observed no clear correlation between overall L1 transposition rates and N_e in chimpanzee (N_e ~ 11,000 [27]), orangutan (N_e ~ 25,000 [28]), or rhesus macaque (N_e ~ 80,000 [29]). Results from population genetics summary statistics

give a similarly mixed picture. Tajima's D, Watterson's Theta, and Pi all suggest elevated diversity and/or reduced directional selection for L1 transduction in chimpanzee, which have the smallest effective population size. However, these statistics show no meaningful difference between orangutan and macaque, two species for which differences in N_e are at least as great as that between orangutan and chimpanzee. Contrasts between the X-chromosome and the autosomes are likewise ambiguous. Because the X chromosome is hemizygous in male primates, deleterious mutations on the X chromosome should be more strongly selected against. And indeed, in orangutan we see proportionally fewer transduction events on the X chromosome as compared to the autosomes ($5.76x10^{-8}$ transductions/bp vs. $6.33x10^{-8}$ transductions/bp). However, the proportions, while lower, are nearly identical between X and autosomes in rhesus macaque ($2.60x10^{-8}$ transductions/bp vs. $2.40\ x10^{-8}$ transductions/bp), a species in which selection should theoretically act more efficaciously. The results together suggest that differences in L1 transduction rates cannot be explained primarily by differences in effective population sizes. Thus, while overall frequencies of TE are dependent on N_e, differential transduction events are more likely to stem from differences in the basic biology of the lineage-specific L1 elements.

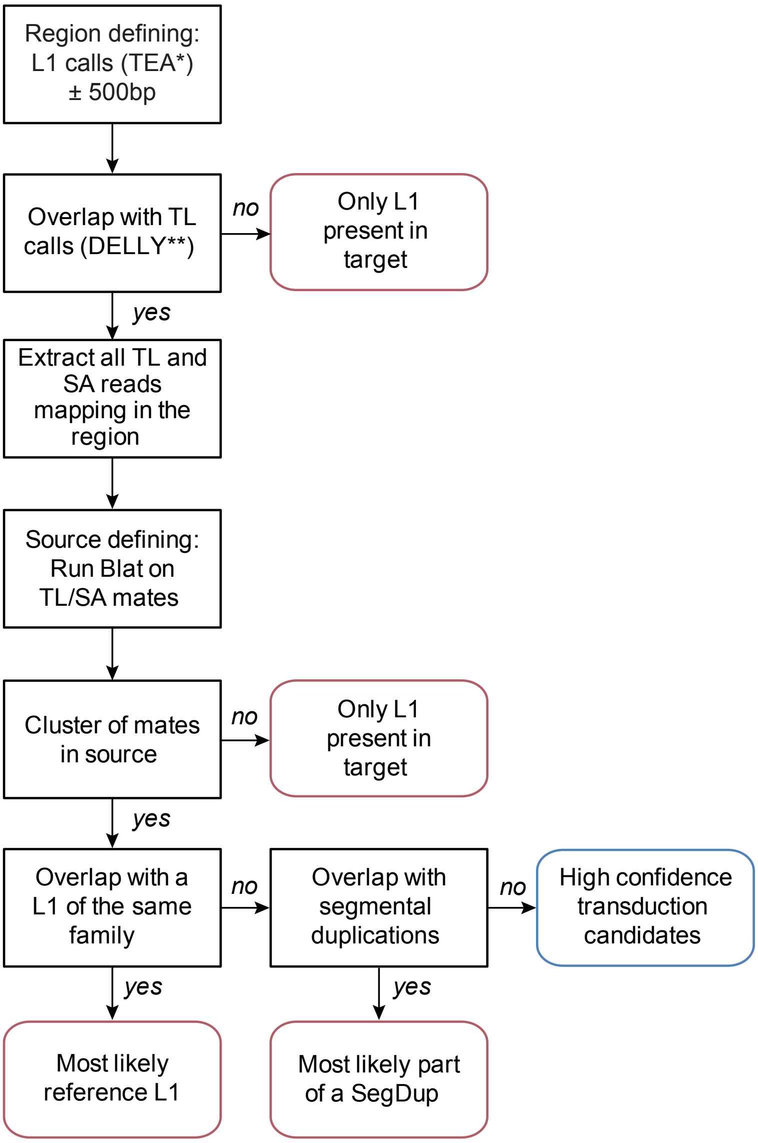

Supplemental Figure 1: TIGER approach. Each L1 coordinate is extended for additional 500 bp (L1 insertion±500bp). If an overlap between this region and at least one translocation (TL) exists, the repetitive L1 element and an additional unique sequence originating from another chromosome are hypothesized to have inserted together, involving an L1-driven transduction event. Once this signature is found and candidate loci are identified, all TL and single-anchored reads (SA reads, mate unmapped or randomly placed in the genome) mapping with one read to the predicted ±500bp surrounding insertion region are obtained from the BAM file and mates are realigned to the corresponding reference genome using the UCSC standalone Blat software. All predicted insertion regions are filtered for overlap with segmental duplications (using the dataset from Gokcumen *et al.* [1]) as well as the presence of a reference L1 at the insertion.

*Lee *et al.* [4]
** Rausch *et al.* [12]

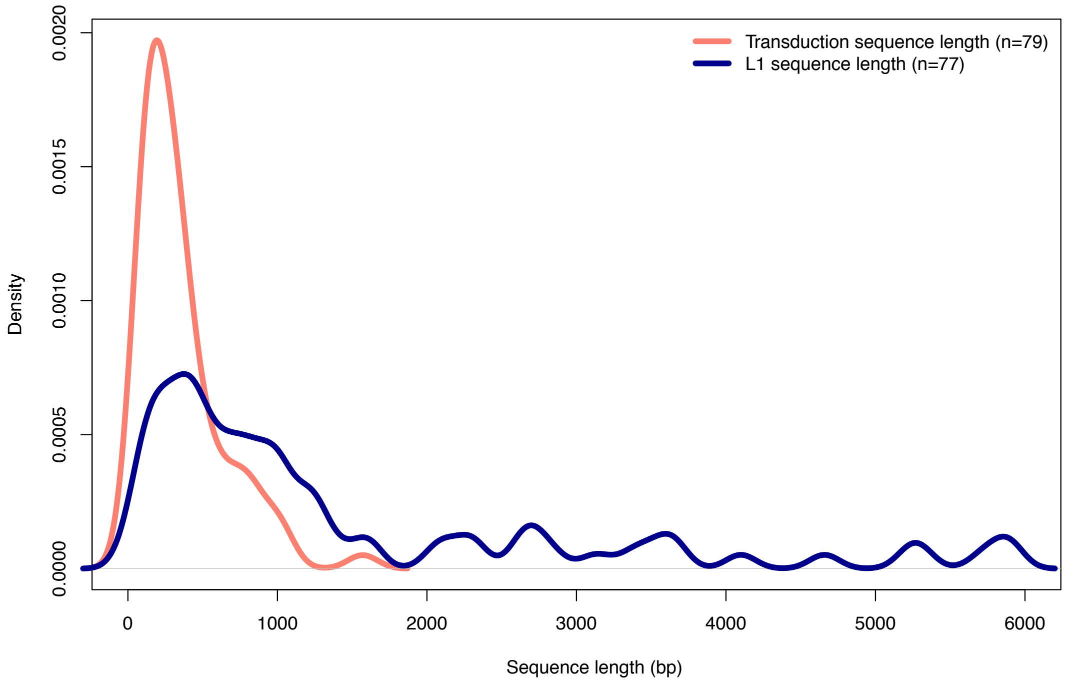

Supplemental Figure 2: Transduction and L1 insertion length based on experimental results. Experimentally determined lengths in three non-human primate species were combined, and ranged from 51 bp - 1570 bp for transductions, whereas L1 lengths were inferred to be up to 6000 bp. This indicates that most of the validated predictions contain severely truncated L1 elements after integration, whereas only a few remaining full-length L1 elements accompany 3' transduction sequences. Note that the size was not always successfully resolved for both the L1 and the transduction part. Values on the y-axis are represented as densities: smoothed lines plotted over histogram with values proportional to the chance that any value in the data is approximately equal to that value.

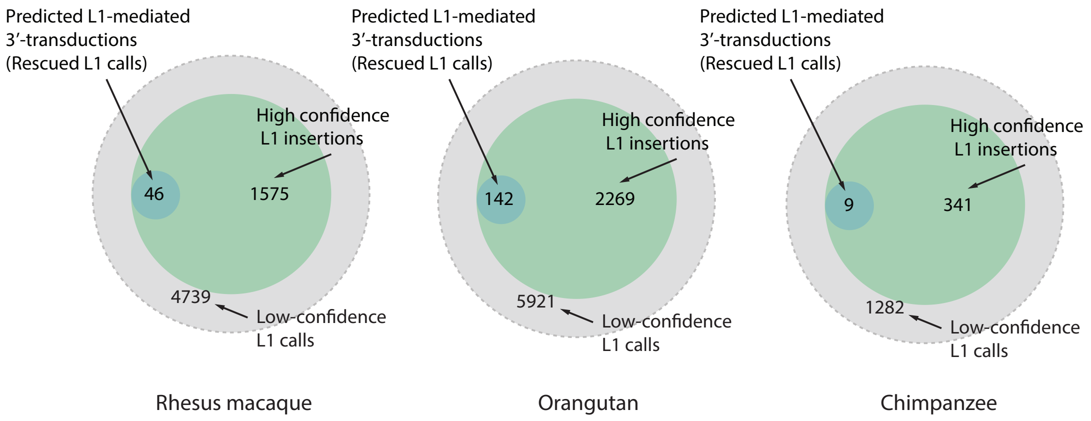

Supplemental Figure 3: Novel L1-transduction calls are contributing to L1 diversity. Previously undetected L1 calls (blue circle) can be rescued by TIGER, which takes low confidence L1 callsets (grey circle) and looks for overlap with translocation calls, resulting in 46, 142 and 9 additional L1 calls detected as transductions in macaque, orangutan and chimpanzee, respectively. These L1 insertions would be lost using standard MEI callers, due to the stringent filtering requiring support for L1 calls on both sides. L1-transduction calls usually have support for the L1 insertion only on one side, whereas on the other side, the unique transduction sequence is supported.

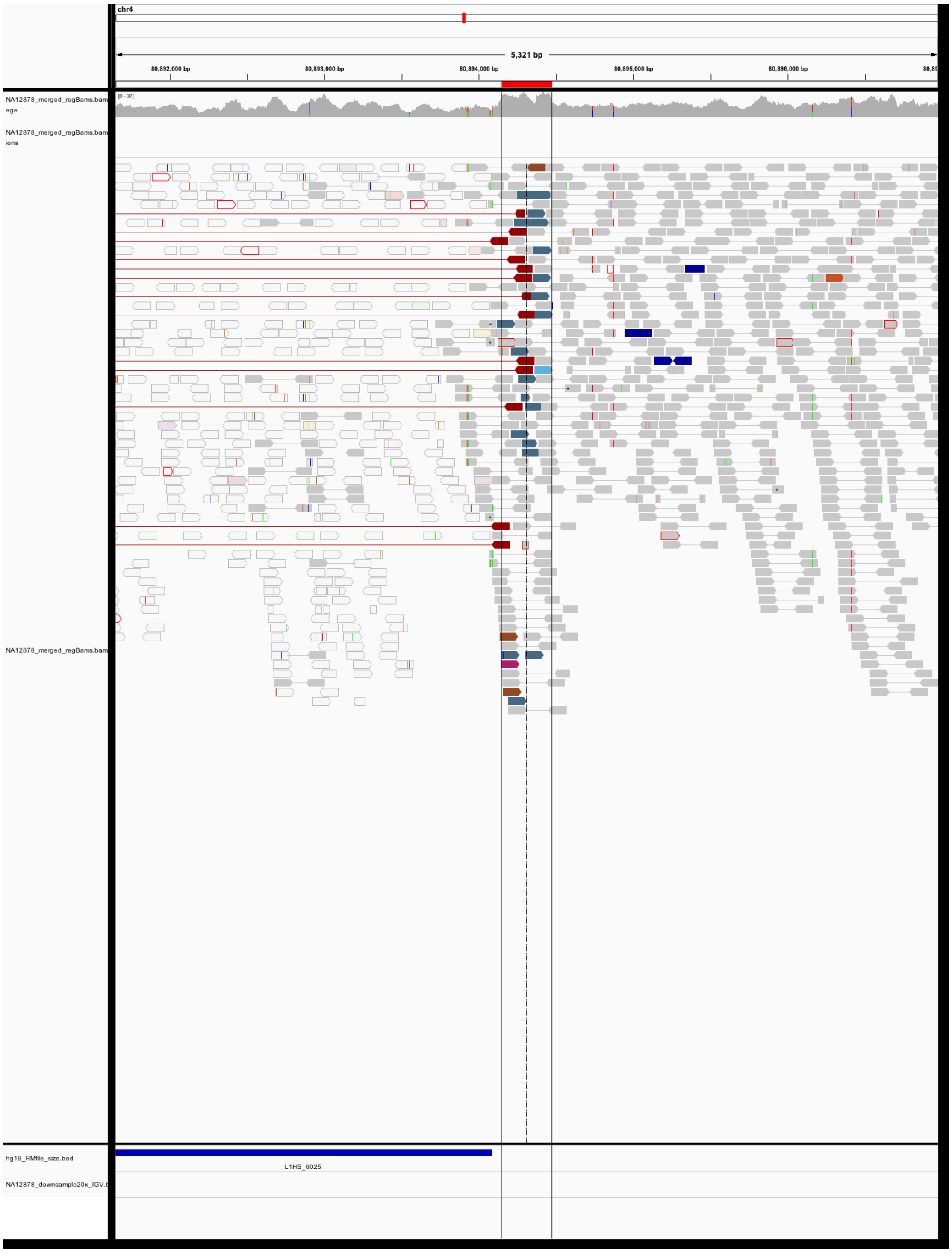
chr4
5,321 bp
80,892,000 bp
80,893,000 bp
80,894,000 bp
80,895,000 bp
80,896,000 bp
NA12878_merged_regBams.bam
age
[0 - 37]
NA12878_merged_regBams.bam
ions
NA12878_merged_regBams.bam
hg19_RMfile_size.bed
L1HS_6025
NA12878_downsample20x_IGV.b

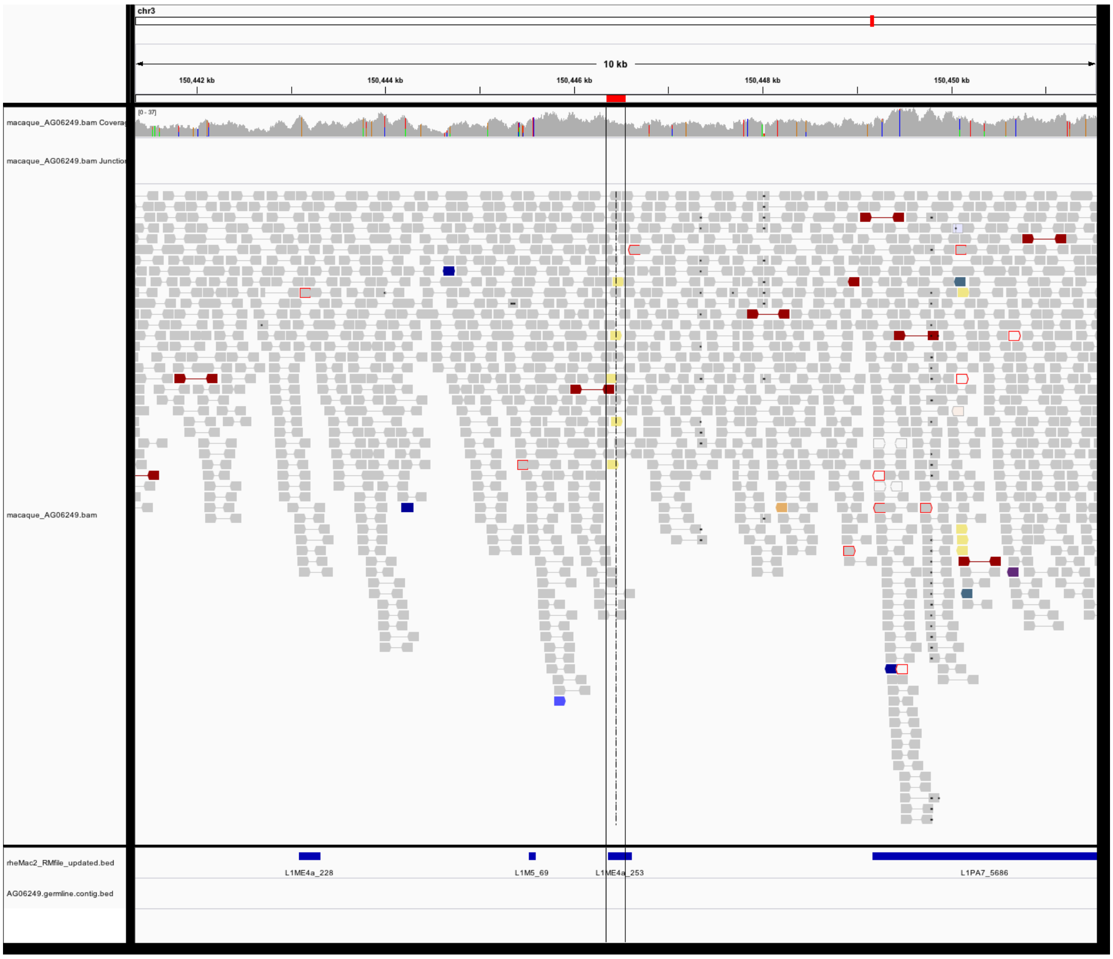

chr3
10 kb
150,442 kb
150,444 kb
150,446 kb
150,448 kb
150,450 kb
macaque_AG06249.bam Coverag
macaque_AG06249.bam Junction
macaque_AG06249.bam
rheMac2_RMfile_updated.bed
AG06249.germline.contig.bed
L1ME4a_228
L1M5_69
L1ME4a_253
L1PA7_5686

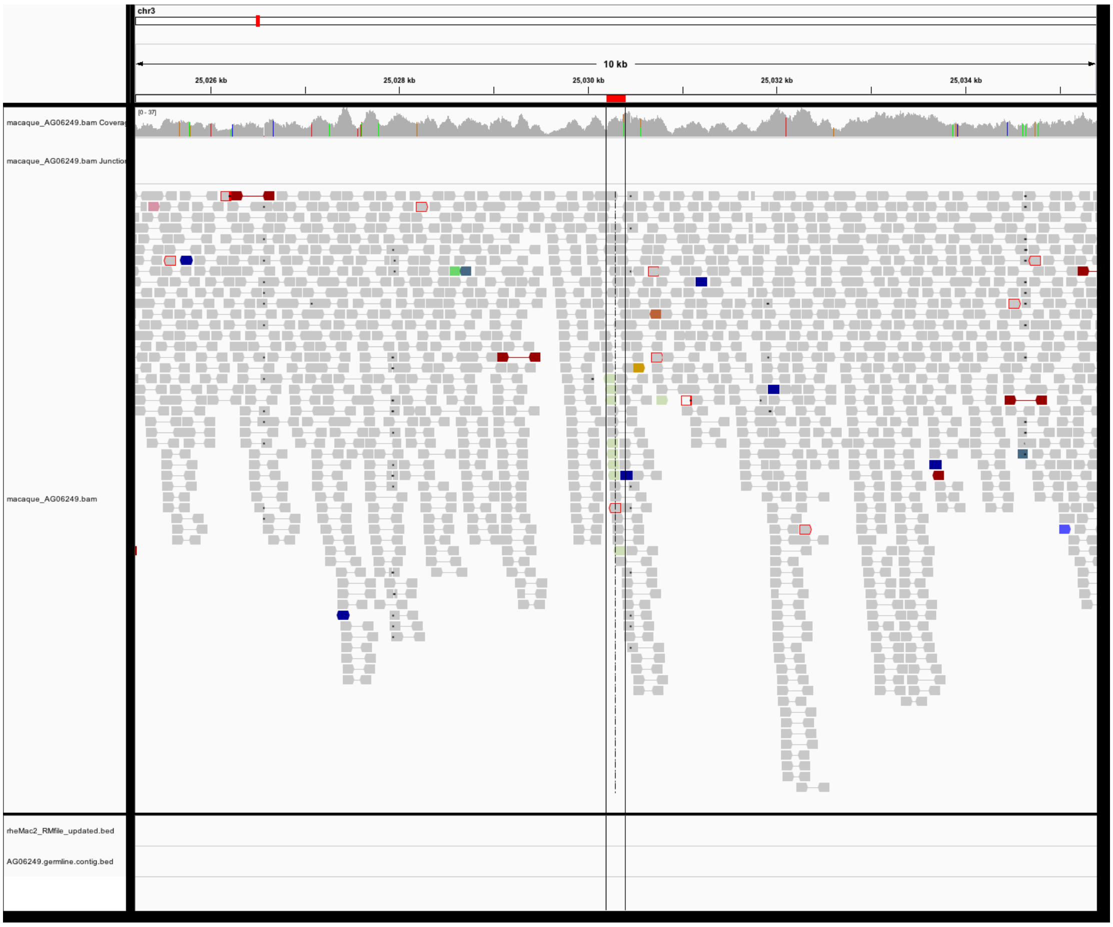
chr3
10 kb
25,026 kb
25,028 kb
25,030 kb
25,032 kb
25,034 kb
[0 - 37]
macaque_AG06249.bam Coverage
macaque_AG06249.bam Junctions
macaque_AG06249.bam
rheMac2_RMfile_updated.bed
AG06249.germline.contig.bed

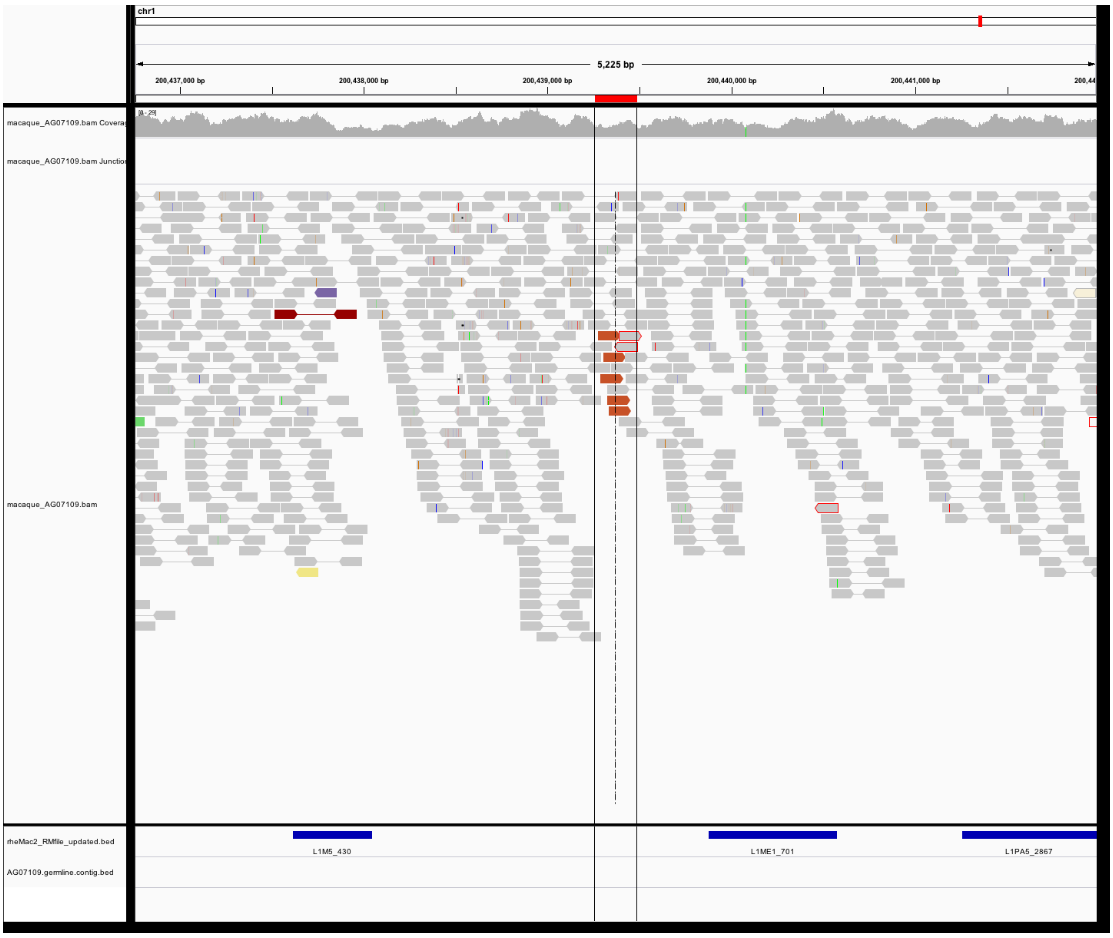

Supplemental Figure 4: IGV examples of source loci in human and rhesus macaque (representation of experimentally validated predictions): The first panel depicts an example of source locus in the human genome NA12878 with a full-length L1 element immediately upstream of transduced sequence (marked in red). The next three panels are all from rhesus macaque: the initial macaque panel represents a single case where there is an L1 element >5 kb in proximity to the predicted source locus (AG06249 individual), followed by the representation of a class 1 element (no donor L1; AG06249 individual) and a class 2 element (small, truncated L1 surrounding the predicted transduction sequence; AG07109 individual). Validated calls were inspected in all individuals (see also Additional data file 4). Numbers listed next to the name of L1 elements show the size (in bp) of each element in a given region.

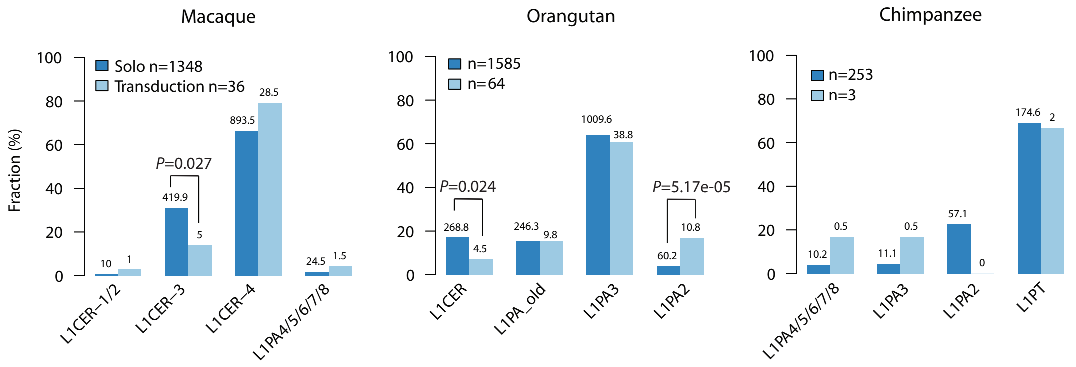

Supplemental Figure 5: Subfamily distribution (multi-annotation) of solo-L1 events in comparison to L1s accompanying transduction sequences. L1s with transduction showed a slightly different subfamily distribution from solo L1s (Fisher's test *P*=0.019 - macaque and *P*=7.496x10^{-5} - orangutan). In macaque, L1CER-4 was enriched in transduction while L1CER-3 was depleted relative to solo-L1s. In orangutan, L1PA2 showed the highest rate of transduction given the abundance of solo L1s (Fisher's test *P*=5.2x10^{-5}). In chimpanzee, there were too few L1 transductions with reliable subfamily annotation.

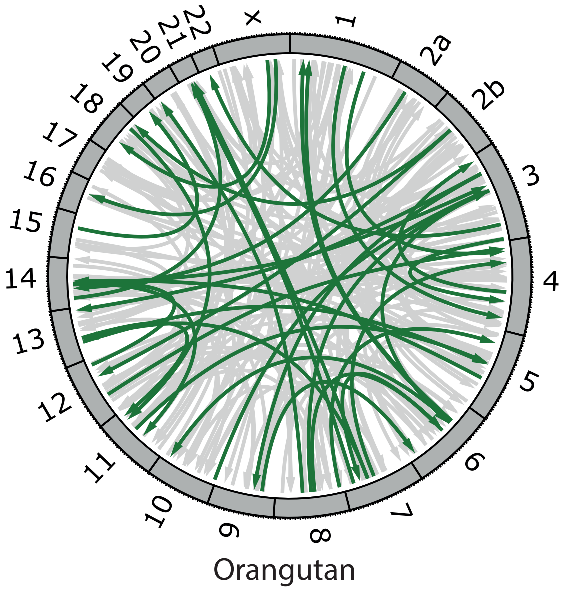

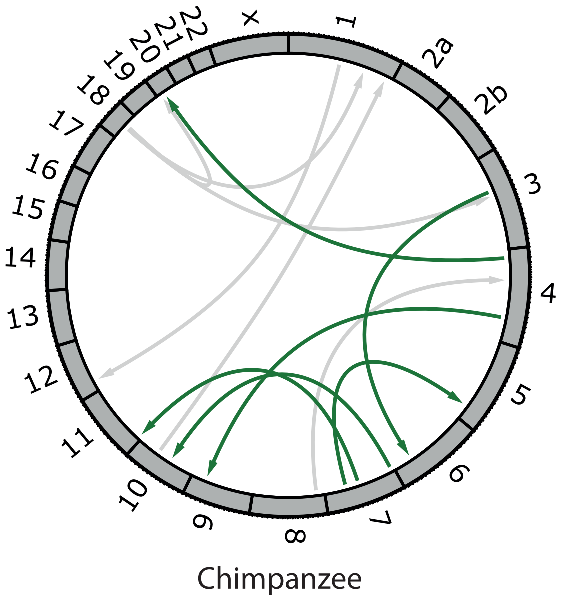

Supplemental Figure 6: Circos plot showing the distribution for all orangutan (upper image) and chimpanzee (lower image) L1-transduction predictions. Experimentally validated (PCR and MinION single-molecule sequencing) insertions in orangutan and chimpanzee, respectively, are depicted in green arrows. Grey arrows: no experimental validation attempted or negative. Arrows indicate the direction of the source inserting into the target locus.

Supplemental Table 1: Human candidate L1 transductions verified either by single molecule sequencing (PacBio) or through presence in previously published data. (Additional data file 2 contains all predicted transduction sequences.)

Target chr	Target start	Target end	Source chr	Source start	Source end	Validation status
chr17	64636774	64637798	chr6	13191202	13191446	Kidd dataset [19]
chr4	104214171	104215187	chr1	81404770	81404952	PacBio
chr9	100675033	100676051	chr1	86398771	86398924	PacBio
chr5	21207213	21208233	chr4	80894147	80894475	Kidd dataset [19]

Supplemental Table 2: Population genetics summary statistics (Tajima's D, Watterson's Theta, and Pi values) for selection effect acting on L1 transductions: The mean of samples as well as the 95% quantiles are recorded.

Species	X_or_autosome	Statistc	Mean	-95%	95%
**Chimpanzee**	Autosomes	Tajima D	0.258317003	-0.850103589	1.410318203
**Chimpanzee**	Autosomes	Theta	0.174680881	0.117828587	0.235657175
**Chimpanzee**	Autosomes	Pi per site	0.183333333	0.112962963	0.22962963
**Orangutan**	X	Tajima D	-0.875737548	-1.667060562	0.473433879
**Orangutan**	X	Theta	0.156319259	0.039276196	0.235657175
**Orangutan**	X	Pi per site	0.119308642	0.022222222	0.192592593
**Orangutan**	Autosomes	Tajima D	-0.680525561	-1.004239301	-0.384519497
**Orangutan**	Autosomes	Theta	0.144870896	0.126244915	0.163147275
**Orangutan**	Autosomes	Pi per site	0.124733822	0.11037851	0.138583639
**Macaque**	X	Tajima D	-0.367969343	-1.400850802	1.302677905
**Macaque**	X	Theta	0.136092019	0	0.265114322
**Macaque**	X	Pi per site	0.114611111	0	0.216666667
**Macaque**	Autosomes	Tajima D	-0.671941901	-1.154216606	-0.133949562
**Macaque**	Autosomes	Theta	0.146617453	0.126621766	0.179380835
**Macaque**	Autosomes	Pi per site	0.125860697	0.104145937	0.15655058

Supplemental Table 3: Dataset adapted from Kidd *et al*., 2010, Cell. Transductions listed are subjected to stringent filtering (described in the table below) to make this dataset comparable to the dataset obtained from the TIGER tool. Last column describes whether TIGER would detect each transduction listed and the corresponding number of transductions detectable with TIGER (in total eight transductions), given that same samples are used.

Acc	hg19-target	hg19-source	Source-size	Comment	Conclusion
AC207587	chr11:40841270-40841289	NA	NA	no source predicted	unable to detect (by TIGER)
AC215798	chr6:108982594-108982609	NA	NA	no source predicted	unable to detect (by TIGER)
AC217325	chr13:41006626-41006644	NA	NA	no source predicted	unable to detect (by TIGER)
AC207451	chr16:71028008-71028024	NA	NA	no source predicted	unable to detect (by TIGER)
AC207594	chr9:5491404-5491419	NA	NA	no source predicted	unable to detect (by TIGER)
AC209305	chr15:35654130-35654145	chr17:68461116-68461173	57	overlaps L1	unable to detect (by TIGER)
AC206473	chr18:12491251-12491263	chrX:11953192-11953399	207	overlaps L1	unable to detect (by TIGER)
AC209011	chr12:34017324-34017339	chr13:48856542-48856830	288	overlaps L1	unable to detect (by TIGER)
AC207969	chr7:8886704-8886716	chr5:24370538-24370797	259	overlaps L1	unable to detect (by TIGER)
AC216813	chr1:179575361-179575378	chrX:94852525-94852552	27	overlaps L1	unable to detect (by TIGER)
AC207983	chr15:56251134-56251153	chr17:68461117-68461177	60	overlaps L1	unable to detect (by TIGER)
AC219161	chr2:144626224-144626236	chr5:24370538-24370797	259	overlaps L1	unable to detect (by TIGER)
AC203662	chr3:20748895-20748905	chr9:12556850-12556937	87	overlaps L1	unable to detect (by TIGER)
AC195775	chr6:72799499-72799516	chr17:68461117-68461178	61	overlaps L1	unable to detect (by TIGER)
AC216122	chr14:31150808-31150827	chr11:24355522-24355568	46	overlaps L1	unable to detect (by TIGER)
AC203592	chr14:52667757-52667768	chr4:170280994-170281923	929	found among TEA calls in our sample, but reads are not unique according to TIGER	unable to detect (by TIGER)
AC214986	chr10:124455216-124455232	chr8:17859502-17859579	77	found among TEA calls in our sample, but reads are not unique according to TIGER	unable to detect (by TIGER)
AC216136	chr4:147141623-147141638	chr1:83127462-83127608	146	would pass the TIGER pipeline	1
AC211854	chr3:38626065-38626083	chr1:83127462-83127575	113	would pass the TIGER pipeline	2
AC208106	chrX:143704668-143704685	chr1:84517937-84518070	133	would pass the TIGER pipeline	3
AC226062	chr3:131069767-131069780	chr16:61079188-61079246	58	would pass the TIGER pipeline	4
AC203635	chr21:23626190-23626206	chr21:29292965-29293560	595	would pass the TIGER pipeline	5
AC208067	chr5:21207713-21207733	chr4:80894075-80894475	400	would pass the TIGER pipeline	6 - also found in our sample
AC209294	chr17:64637274-64637295	chr6:13191016-13191446	430	would pass the TIGER pipeline	7 - also found in our sample
AC206439	chr9:7741396-7741407	chr6:16862322-16862389	67	would pass the TIGER pipeline	8

Supplemental Table 4: Gene Retrocopy Insertion Polymorphisms (GRIPs). Two loci predicted to contain unique sequence insertion mediated by the L1-machinery (one in orangutan and one in rhesus macaque) were experimentally verified to underlie GRIPs.

Species	Target	Source	Gene	Experimentally validated
**Orangutan**	chr6:80007306-80007324	chr11:81410251-81410341	TMEM126B	Yes
**Macaque**	chr7:65507000 -65507014	chr1:42440379-42440521	PABPC4/5	Yes

Supplemental Table 5: Comparison of 3' transduction rates (%). Numbers listed in the parenthesis are representing transduction rates reported in original studies, other values are adjusted based on our analyses to make them comparable to our study.

Macaque germline TS*	Orangutan germline TS*	Chimpanzee germline TS*	Human germline TS*	Human germline TS**	Human somatic partnered TS***
5.5±1.2	8.8±1.4	2.5±1.1	7.5	10.8 (20)	11.2 (22.4)

*Determined using TIGER approach
**Adapted from Kidd *et al.*– with comparable parameters used in TIGER (calls predicted to insert into L1s were removed, repetitive transduced sequence and transductions with no source predicted were also removed)
***Adapted from Tubio *et al.*– only partnered L1-mediated transductions with comparable parameters used in TIGER (calls predicted to insert into L1s were removed)

REFERENCES:

1. Gokcumen O, Tischler V, Tica J, Zhu Q, Iskow RC, Lee E, Fritz MH, Langdon A, Stutz AM, Pavlidis P, et al: **Primate genome architecture influences structural variation mechanisms and functional consequences.** *Proc Natl Acad Sci U S A* 2013, **110:**15764-15769.
2. The 1000 Genomes Project Consortium, Abecasis GR, Altshuler D, Auton A, Brooks LD, Durbin RM, Gibbs RA, Hurles ME, McVean GA: **A map of human genome variation from population-scale sequencing.** *Nature* 2010, **467:**1061-1073.
3. Li H, Durbin R: **Fast and accurate short read alignment with Burrows-Wheeler transform.** *Bioinformatics* 2009, **25:**1754-1760.
4. Lee E, Iskow R, Yang L, Gokcumen O, Haseley P, Luquette LJ, 3rd, Lohr JG, Harris CC, Ding L, Wilson RK, et al: **Landscape of somatic retrotransposition in human cancers.** *Science* 2012, **337:**967-971.
5. Tubio JM, Li Y, Ju YS, Martincorena I, Cooke SL, Tojo M, Gundem G, Pipinikas CP, Zamora J, Raine K, et al: **Mobile DNA in cancer. Extensive transduction of nonrepetitive DNA mediated by L1 retrotransposition in cancer genomes.** *Science* 2014, **345:**1251343.
6. Quinlan AR, Hall IM: **BEDTools: a flexible suite of utilities for comparing genomic features.** *Bioinformatics* 2010, **26:**841-842.
7. Li H, Handsaker B, Wysoker A, Fennell T, Ruan J, Homer N, Marth G, Abecasis G, Durbin R, Genome Project Data Processing S: **The Sequence Alignment/Map format and SAMtools.** *Bioinformatics* 2009, **25:**2078-2079.
8. Kent WJ: **BLAT--the BLAST-like alignment tool.** *Genome Res* 2002, **12:**656-664.
9. Wildschutte JH, Baron A, Diroff NM, Kidd JM: **Discovery and characterization of Alu repeat sequences via precise local read assembly.** *Nucleic Acids Res* 2015, **43:**10292-10307.
10. Helman E, Lawrence MS, Stewart C, Sougnez C, Getz G, Meyerson M: **Somatic retrotransposition in human cancer revealed by whole-genome and exome sequencing.** *Genome Res* 2014, **24:**1053-1063.
11. Solyom S, Ewing AD, Rahrmann EP, Doucet T, Nelson HH, Burns MB, Harris RS, Sigmon DF, Casella A, Erlanger B, et al: **Extensive somatic L1 retrotransposition in colorectal tumors.** *Genome Res* 2012, **22:**2328-2338.
12. Rausch T, Zichner T, Schlattl A, Stutz AM, Benes V, Korbel JO: **DELLY: structural variant discovery by integrated paired-end and split-read analysis.** *Bioinformatics* 2012, **28:**i333-i339.
13. Huang X, Madan A: **CAP3: A DNA sequence assembly program.** *Genome Res* 1999, **9:**868-877.
14. Han K, Konkel MK, Xing J, Wang H, Lee J, Meyer TJ, Huang CT, Sandifer E, Hebert K, Barnes EW, et al: **Mobile DNA in Old World monkeys: a glimpse through the rhesus macaque genome.** *Science* 2007, **316:**238-240.

15. Untergasser A, Nijveen H, Rao X, Bisseling T, Geurts R, Leunissen JA: **Primer3Plus, an enhanced web interface to Primer3.** *Nucleic Acids Res* 2007, **35:**W71-74.
16. Venables WN, Ripley BD: *Modern Applied Statistics with S.* Springer New York; 2002.
17. Dunn P, Smyth G: **Series evaluation of Tweedie exponential dispersion model densities.** *Statistics and Computing* 2005, **15:**267-280.
18. Bretz F, Hothorn T, Westfall P: *Multiple Comparisons Using {R}.* Boca Raton, Florida, USA: Chapman & Hall/CRC Press; 2010.
19. Kidd JM, Graves T, Newman TL, Fulton R, Hayden HS, Malig M, Kallicki J, Kaul R, Wilson RK, Eichler EE: **A human genome structural variation sequencing resource reveals insights into mutational mechanisms.** *Cell* 2010, **143:**837-847.
20. Fujita PA, Rhead B, Zweig AS, Hinrichs AS, Karolchik D, Cline MS, Goldman M, Barber GP, Clawson H, Coelho A, et al: **The UCSC Genome Browser database: update 2011.** *Nucleic Acids Res* 2011, **39:**D876-882.
21. Hazzouri KM, Mohajer A, Dejak SI, Otto SP, Wright SI: **Contrasting patterns of transposable-element insertion polymorphism and nucleotide diversity in autotetraploid and allotetraploid Arabidopsis species.** *Genetics* 2008, **179:**581-592.
22. Garfield D, Haygood R, Nielsen WJ, Wray GA: **Population genetics of cis-regulatory sequences that operate during embryonic development in the sea urchin Strongylocentrotus purpuratus.** *Evol Dev* 2012, **14:**152-167.
23. Kidwell MG, Lisch DR: **Perspective: transposable elements, parasitic DNA, and genome evolution.** *Evolution* 2001, **55:**1-24.
24. Brookfield JF: **The ecology of the genome - mobile DNA elements and their hosts.** *Nat Rev Genet* 2005, **6:**128-136.
25. Charlesworth B, Sniegowski P, Stephan W: **The evolutionary dynamics of repetitive DNA in eukaryotes.** *Nature* 1994, **371:**215-220.
26. Bulmer M: **The selection-mutation-drift theory of synonymous codon usage.** *Genetics* 1991, **129:**897-907.
27. Auton A, Fledel-Alon A, Pfeifer S, Venn O, Segurel L, Street T, Leffler EM, Bowden R, Aneas I, Broxholme J, et al: **A fine-scale chimpanzee genetic map from population sequencing.** *Science* 2012, **336:**193-198.
28. Locke DP, Hillier LW, Warren WC, Worley KC, Nazareth LV, Muzny DM, Yang SP, Wang Z, Chinwalla AT, Minx P, et al: **Comparative and demographic analysis of orang-utan genomes.** *Nature* 2011, **469:**529-533.
29. Yuan Q, Zhou Z, Lindell SG, Higley JD, Ferguson B, Thompson RC, Lopez JF, Suomi SJ, Baghal B, Baker M, et al: **The rhesus macaque is three times as diverse but more closely equivalent in damaging coding variation as compared to the human.** *BMC Genet* 2012, **13:**52.
